# N-Glycanase 1 Deficiency Is a Rare Cause of Pediatric Neurodegeneration With Neuronal Inclusions and Liver Steatosis

**DOI:** 10.7759/cureus.19126

**Published:** 2021-10-29

**Authors:** Thomas Stuut, Oana Popescu, Angelica Oviedo

**Affiliations:** 1 Family Medicine, Central Michigan University College of Medicine, Mount Pleasant, USA; 2 Pathology and Laboratory Medicine, Vernon Jubilee Hospital, Vernon, CAN; 3 Pathology and Laboratory Medicine, Burrell College of Osteopathic Medicine, Las Cruces, USA

**Keywords:** pediatric neurodegeneration, liver steatosis, neuronal inclusion, developmental delay, myoclonic epilepsy, n-glycanase 1 deficiency

## Abstract

Pediatric neurodegeneration is extremely rare and devastating to the families involved. We describe a rare case of pediatric neurodegeneration in a child with N-glycanase 1 (NGLY1) deficiency. This child had an autosomal recessive mutation in *NGLY1*, the gene coding for the enzyme NGLY1 that was found with exome sequencing. NGLY1 catalyzes protein deglycosylation by cleaving the -aspartyl glycosylamine bond of N-linked glycoproteins and is thereby a component of the endoplasmic reticulum-associated degradation pathway. This child passed away at five years of age after a prolonged clinical course with myoclonic epilepsy, choreoathetosis-like movements, lacrimal duct problems, and severe developmental delay. This autopsy case report shows images of the neuronal inclusions and liver steatosis found in this patient with NGLY1 deficiency and offers a detailed clinical history.

## Introduction

We report the detailed clinical history and autopsy findings of a rare case of pediatric neurodegeneration caused by autosomal recessive inheritance of a mutation in *NGLY1*, the gene coding for the enzyme N-glycanase 1 (NGLY1). NGLY1 catalyzes protein deglycosylation by cleaving the -aspartyl glycosylamine bond of N-linked glycoproteins. Studies of NGLY1 across various species suggest it is a key cytoplasmic component of the endoplasmic reticulum-associated degradation (ERAD) pathway, which is a mechanism for identifying and degrading misfolded glycoproteins. Loss of NGLY1 would be expected to result in misfolded protein accumulation due to impaired degradation; it has also been shown to cause decreased mitochondrial function [1]. Our patient was part of a large whole-exome Sanger (WES) sequencing research study in patients with undiagnosed genetic conditions; that study identified eight patients with NGLY1 deficiency due to autosomal recessive inheritance of *NGLY1* mutation [2]. Prior to this large study, one patient with NGLY1 deficiency had been identified, also by WES [3]. All patients identified to date have a syndrome characterized by severe complex CNS involvement along with changes in other organ systems. Our patient passed away at five years of age, following a viral illness and prolonged seizures. He underwent extensive diagnostic studies during life. Autopsy demonstrated neuronal inclusions secondary to the misfolded protein accumulation, and liver steatosis secondary to NGLY1 deficiency. Although WES is invaluable in diagnoses of these rare syndromes, we offer a detailed clinical history and pathologic findings in order to correlate the NGLY1 deficiency with morphologic findings in this rare disease.

## Case presentation

This five-year-old boy had a history of long-standing neurodegenerative disease with myoclonic epilepsy, choreoathetosis-like movements, lacrimal duct problems, and severe developmental delay. The mother had a positive second-trimester screen for trisomy 18 and Smith-Lemli-Opitz syndrome with alpha-fetoprotein (AFP) 1.97 multiple of the median (MoM), uE3 0.24 MoM, and hCG 0.48 MoM. At 36-weeks, the mother underwent a Cesarean section for findings of intrauterine growth restriction and oligohydramnios. At birth, the neonate had a length of 44 cm (10th percentile), a weight of 1886 g (3rd percentile), and a head circumference of 32 cm (10th percentile). He was noted to have mild flexion contractures at the knees and a weak suck and was hospitalized for 11 days secondary to poor feeding. At eight months, his liver transaminase levels were found to be elevated (ALT and AST twice normal with GGT four times normal) and the elevation persisted at least until he was three and a half years old. Despite normal-appearing deep tendon reflexes in infancy, by two years of age, his reflexes were diminished and by 38 months, they were no longer present. By 36 months, he developed myoclonic seizures that persisted despite treatment. The patient also had global developmental delay [2]. At the age of five years, after developing seizures and a temperature reaching as high as 42.3 °C, the patient died.

**Figure 1:**
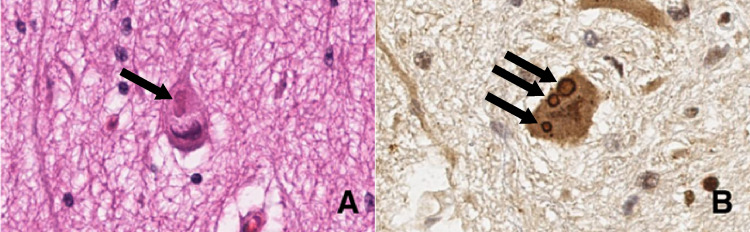
Intraneuronal inclusions anterior horn spinal cord motor neurons. Anterior horn spinal cord motor neuron cytoplasmic inclusions visualized with hematoxylin and eosin stain (A) and ubiquitin immunohistochemistry staining (B). Ubiquitin staining demonstrates involvement of the endoplasmic reticulum-associated degradation pathway and accumulation of misfolded proteins.

On autopsy, the brain weighed 1115 g, which was considered within normal limits for age. Histopathology showed the presence of eosinophilic cytoplasmic inclusions in the globus pallidus, red nucleus, subthalamic nucleus, dentate nucleus, spinal cord, cortex, and dorsal root ganglion. The spinal cord neuronal inclusions are shown (Figure 1). Glial inclusions were not seen. There was also extensive cerebellar dentate nucleus and Purkinje cell loss. The brain also showed extensive hypoxic-ischemic changes considered end-stage changes. 

**Figure 2:**
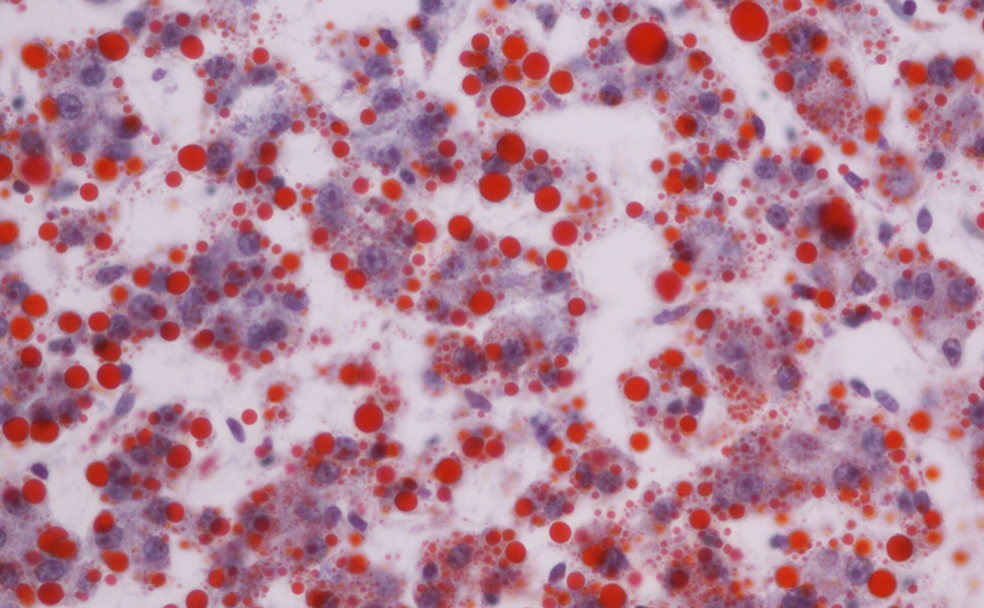
Liver steatosis. Oil-red-O staining shows hepatocytes with marked periportal and perivenular micro and macro-vesicular steatosis.  [This image was brightened with Adobe Photoshop (Adobe Inc. San Jose, California)].

Additional autopsy findings included an enlarged liver with hepatocytes showing marked periportal and perivenular micro and macro-vesicular steatosis, confirmed with Oil-red-O staining (Figure 2).

This patient was part of a whole exome sequencing research study, which showed the presence of a homozygous, single nucleotide variant N-glycanase 1 (NGLY1) enzyme mutation resulting from a T to A switch at position 3:25775422 (hg19), which causes a nonsense mutation, R401X [2]. The parents were non-consanguineous and both were found to be heterozygous for this mutation.

## Discussion

Our case study highlights the detailed clinical history and autopsy findings of this patient with NGLY1 deficiency. Given its role in the cell, a deficiency of NGLY1 would be expected to result in the accumulation of misfolded glycoproteins due to impaired degradation; this is seen morphologically in our patient as intraneuronal inclusions. NGLY1 deficiency has also been directly linked to decreased mitochondrial function [1], which has been implicated in hepatic steatosis secondary to decreased fatty acid oxidation [4]. The liver steatosis in our patient is considered secondary to his NGLY1 deficiency and resulting decreased mitochondrial function. No other inclusions or abnormal cytoplasmic accumulations were seen in other organ systems.

The first identified NGLY1 deficient patient had a history of developmental delay, multifocal epilepsy, involuntary movements, abnormal liver function, and absent tears [3]; this history is almost identical to our patient’s history. Also, the liver biopsy from this first identified NGLY1 deficient patient was described as containing an “amorphous unidentified substance” in the cytoplasm [3]; however, no further characterization was described, and no liver images were shown. Our patient’s liver had Oil-red-O positive droplets, suggesting that the unidentified substance in the previous patient’s liver was likely lipid due to decreased mitochondrial function. There were seven additional patients with NGLY1 deficiency described in the paper that described the NGLY1 mutation in our patient [2]. Five of these patients had the same homozygous R401X nonsense mutation; one patient had a homozygous R524X nonsense mutation while one patient had a homozygous frameshift R458fs mutation. The nonsense mutations would be expected to produce no functional NGLY1 enzyme while the frameshift mutation resulted in a stop codon at the end of exon 9 and also likely did not have functional NGLY1 (that patient was heavily affected clinically). Overall, the previously reported patients demonstrated similar clinical histories of abnormal tear production, choreoathetosis, and liver disease, which were also present in our patient. Other commonly reported abnormalities also present in our patient included intrauterine growth restriction, developmental delay, and seizures.

## Conclusions

We present this case of *NGLY1* autosomal recessive mutation in order to show the associated pathology with neuronal cytoplasmic inclusions in the brain and spinal cord along with steatosis in the liver. In addition, this case is an example of the value of whole-exome sequencing for rare diseases, since rare genes may not be part of the common gene panels used. The detailed clinical history will allow healthcare providers to recognize this rare entity during life. It is important to recognize these rare syndromes for future family planning and so that families understand the disease that has affected their child. It is also important to correlate the genetic findings with morphologic findings in these rare diseases, so that they may be further characterized, and potential therapies may be found.
